# Structured reporting in neuro-oncological imaging: achieving reliable prediction of molecular subtypes in glioma based on pre-treatment multi-sequence MRI

**DOI:** 10.1007/s00330-021-08210-3

**Published:** 2021-08-07

**Authors:** Nico Sollmann

**Affiliations:** 1 Department of Diagnostic and Interventional Radiology, University Hospital Ulm, Albert-Einstein-Allee 23, 89081 Ulm, Germany; 2 Department of Diagnostic and Interventional Neuroradiology, School of Medicine, Klinikum rechts der Isar, Technical University of Munich, Ismaninger Str. 22, 81675 Munich, Germany; 3 TUM-Neuroimaging Center, Klinikum rechts der Isar, Technical University of Munich, Munich, Germany

Structured reporting commonly refers to the use of predefined formats and terms to create radiological reports, using a high level of standardized and organized information in template context [1, 2]. In a recent publication, the European Society of Radiology (ESR) proposed the triad of quality, datafication/quantification, and accessibility as a major requirement for the transition from conventional “free-text” reporting to structured reporting in radiology [3]. First, improvement of quality for radiological reporting may be achieved through standardization, which can be established by implementation of templates that provide a sort of checklist of items that need to be included in the report, commonly in a predefined order. Second, datafication/quantification refers to the inclusion of specific data elements (e.g., grading systems like the TNM classification) and quantitative parameters (e.g., radiomics or image-based biomarkers) in the report. Third, availability plays an essential role for broad consideration of radiological information with multifarious purposes including diagnosis, clinical decision-making, and transfer in order to target research questions or generate representative databases for specific pathologies or imaging findings.

The study by Nam et al [4], published in *European Radiology*, aims at identifying reproducible parameters derived from cranial magnetic resonance imaging (MRI) that are predictive of molecular subtypes in glioma using a structured reporting system. Specifically, in this structured reporting system, MRI-derived parameters including tumor necrosis and margin, edema, internal cyst, T2/fluid-attenuated inversion recovery (FLAIR) mismatch, contrast enhancement patterns, and cerebral blood volume (CBV) higher than normal cortex were considered, which have been derived from multi-sequence scanning with non-contrast and contrast-enhanced T1- and T2-weighted, and FLAIR sequences as well as CBV maps. These image datasets were visually analyzed, and structured reports were created by three neuroradiologists with different experience levels (1 to 7 years) in a training set of 141 and a validation set of 131 patients with glioma. Included patients were stratified into five risk types considering pathomolecular evaluation with isocitrate dehydrogenase (IDH) mutation and 1p19q co-deletion status of glioma, similar to the risk types proposed previously [5]. The main finding is that in the validation set, the prediction of three of the five defined risk types showed high diagnostic performance regarding predominant enhancement (for IDH-wildtype glioblastoma), T2/FLAIR mismatch sign and no necrosis (for IDH-mutant diffuse astrocytoma, grades II/III), and internal cyst or necrosis (for IDH-mutant oligodendroglioma, 1p19q co-deleted). Thus, by means of structured reporting, the study was successful in identifying imaging parameters in pre-treatment MRI that would facilitate classification of some glioma entities. Of note, diagnostic modeling to achieve these results considered the MRI-based parameters that achieved an agreement of > 50% among the three readers, and final diagnosis was based on pathomolecular evaluation using tumor tissue probes.

This study represents an appealing way to implement structured reporting to address a common diagnostic issue in neuroradiology. Extracting parameters from pre-treatment MRI acquisitions has the potential to facilitate early non-invasive diagnosis and tailored treatment decision-making. Specifically, it has been suggested previously that treatment approaches may be associated with certain characteristics observed in pre-treatment MRI of glioma, with the choice of neurosurgical intervention being significantly related to the complexity of tumor infiltration and contrast enhancement patterns since patients whose lesions had indiscernible contrast enhancement received a more conservative management by approximately three times longer intervals between MRI acquisitions and surgery [6]. In this regard, follow-up studies may extend the present work of Nam et al [4] by also including further sequences that are increasingly applied in clinical imaging protocols, such as diffusion tensor imaging (DTI) or alternative T1-weighted sequences to standardly used turbo field-echo imaging. Specifically, DTI may further facilitate discrimination between tumor entities according to IDH status since DTI-derived fractional anisotropy (FA) values were significantly different between oligodendroglial tumors with IDH mutations and those without mutations, for instance [7, 8]. Furthermore, implementation of 3D black-blood turbo spin-echo sequences could facilitate clear depiction of tumor-related contrast enhancement, tumor borders, and satellite lesions that are not or only inferiorly visualized in conventional T1-weighted imaging [9, 10].

In agreement with the recommendations of the ESR, the present study by Nam et al [4] comprehensively used structured reporting that fulfills the criteria of quality through standardization and accessibility, with the structured reports being the basis of predictive modeling targeting glioma stratification. With respect to datafication/quantification, clear categories for structured reporting within a template have been defined by the authors. Beyond the scope of this study is the additional use of quantitative MRI-based data, which is commonly not extracted and not routinely available in the current clinical setup for neuro-oncological imaging. The next step based on this study’s promising results and also similar studies for other body regions would be to accomplish seamless availability of quantitative parameters for structured reporting in the routine setup. Here, both increasingly used sequences that allow extraction of meaningful values (e.g., DTI with extraction of FA or other scalar measures) as well as radiomics could ideally refine pre-treatment MRI-based prediction of tumor entities in the future, supplementing standardized visual image reading by objective metrics from quantitative MRI. However, besides achieving a workable solution that effectively combines visual image reading with quantitative parameters, integration into the radiologic workflow and existing technical infrastructure is key for practicability in daily clinical routine. Once further steps of integration have been taken, structured reporting fusing visual image reading with quantitative parameters certainly has high potential to considerably advance radiological reporting, and to get far more information out of contemporary MRI acquisitions.

In summary, the work by Nam et al [4] comes timely and can be interpreted as a clear advocate for speeding up integration of structured reporting into the clinical workflow, given this study’s comprehensible results based on pre-treatment MRI that may, at least for some glioma entities, augur correct tumor classification with reasonable diagnostic accuracy. The consideration of independent training and validation datasets as well as imaging parameters that adhere to a certain threshold of inter-reader agreement speak for the robustness and quality of this study.
